# Error Propagation in Isometric Log-ratio Coordinates for Compositional Data: Theoretical and Practical Considerations

**DOI:** 10.1007/s11004-016-9646-x

**Published:** 2016-07-07

**Authors:** Mehmet Can Mert, Peter Filzmoser, Karel Hron

**Affiliations:** 1 Institute of Statistics and Mathematical Methods in Economics, Vienna University of Technology, Wiedner Hauptstrasse 8-10, 1040 Vienna, Austria; 2 Department of Mathematical Analysis and Applications of Mathematics, Faculty of Science, Palacký University, 17. listopadu 12, 771 46 Olomouc, Czech Republic

**Keywords:** Aitchison geometry, Orthonormal coordinates, Taylor approximation, Compositional differential calculus, Detection limit

## Abstract

Compositional data, as they typically appear in geochemistry in terms of concentrations of chemical elements in soil samples, need to be expressed in log-ratio coordinates before applying the traditional statistical tools if the relative structure of the data is of primary interest. There are different possibilities for this purpose, like centered log-ratio coefficients, or isometric log-ratio coordinates. In both the approaches, geometric means of the compositional parts are involved, and it is unclear how measurement errors or detection limit problems affect their presentation in coordinates. This problem is investigated theoretically by making use of the theory of error propagation. Due to certain limitations of this approach, the effect of error propagation is also studied by means of simulations. This allows to provide recommendations for practitioners on the amount of error and on the expected distortion of the results, depending on the purpose of the analysis.

## Introduction

Compositional data analysis is concerned with analyzing the relative information between the variables, the so-called compositional parts, of a multivariate data set. Here, relative information refers to the log-ratio methodology (Aitchison 1986) and, therefore, in fact, to an analysis of logarithms of ratios between the compositional parts. It has been demonstrated that the sample space of compositions is not the usual Euclidean space, but the simplex with the so-called Aitchison geometry (Pawlowsky-Glahn et al. 2015). For a composition $\varvec{x}=(x_1, \ldots , x_D)$ with *D* parts, the simplex sample space is defined as

$$\begin{aligned} \mathcal {S}^D=\{\varvec{x}=(x_1,\ldots ,x_D)\quad \text{ such } \text{ that } x_j>0 ~\forall j,\quad \sum _{j=1}^Dx_j=\kappa \} \end{aligned}$$

for an arbitrary constant $\kappa$. Nevertheless, according to recent developments, the sample space of compositional data is even more general (Pawlowsky-Glahn et al. 2015): A vector $\varvec{x}$ is a *D*-part composition when all its components are strictly positive real numbers and carry only relative information. Note that the term relative information is equivalent to information lies in the ratios between the components, not in the absolute values. As a consequence, the actual sample space of compositional data is formed by equivalence classes of proportional positive vectors. Therefore, any constant sum constraint is just a proper representation of compositions that honors the scale invariance principle of compositions: the information in a composition does not depend on the particular units, in which the composition is expressed (Egozcue 2009). In practical terms, the choice of the constant $\kappa$ is irrelevant, since it does not alter the results from a log-ratio-based analysis. In that sense, a discussion on whether the values of an observation sum up to the same constant is needless, this would not make any difference for the analysis considered in this paper. Though for the purpose of better interpretability or visualization, one could also express compositions with the closure operator

$$\begin{aligned} \mathcal {C}(\varvec{x})= \left( \frac{\kappa x_1}{\sum _{j=1}^Dx_j}, \ldots , \frac{\kappa x_D}{\sum _{j=1}^Dx_j} \right) , \end{aligned}$$

which, then, sum up to the constant $\kappa$.

The Aitchison geometry defines a vector space structure of the simplex by the basic operations of perturbation and powering. Given two compositions $\varvec{x}=(x_1, \ldots , x_D)$ and $\varvec{y}=(y_1, \ldots , y_D)$ in $\mathcal {S}^D$, perturbation refers to vector addition, and is defined as

$$\begin{aligned} \varvec{x}\oplus \varvec{y}= \mathcal {C}(x_1y_1, \ldots , x_Dy_D). \end{aligned}$$

Powering refers to a multiplication of a composition $\varvec{x}=(x_1, \ldots , x_D) \in \mathcal {S}^D$ by a scalar $\alpha \in {{\mathbb {R}}}$, and is defined as

$$\begin{aligned} \alpha \odot \varvec{x}=\mathcal {C}(x_1^{\alpha },x_2^{\alpha },\ldots ,x_D^{\alpha }). \end{aligned}$$

Furthermore, the Aitchison inner product, the Aitchison norm, and the Aitchison distance have been defined, and they lead to a Euclidean vector space structure (Pawlowsky-Glahn et al. 2015). All these definitions employ log-ratios between the compositional parts; for instance, the Aitchison inner product between the compositions $\varvec{x}$ and $\varvec{y}$ is given as

$$\begin{aligned} \langle \varvec{x},\varvec{y}\rangle _A=\frac{1}{2D}\sum _{j=1}^D\sum _{k=1}^D\ln \frac{x_j}{x_k}\ln \frac{y_j}{y_k}, \end{aligned}$$

that leads to the Aitchison norm and distance

$$\begin{aligned} ||\varvec{x}||_A=\sqrt{\langle \varvec{x},\varvec{x}\rangle _A},\quad d_A(\varvec{x},\varvec{y})=||\varvec{x}\oplus (-1)\odot \varvec{y}||_A \end{aligned}$$

respectively. Working directly in the simplex sample space is not straightforward. Rather, it is common to express compositional data in the usual Euclidean geometry. In the literature, one frequently refers to transformations; here, it is prefered to use the terminology of expressing the compositions in appropriate coordinates with respect to the Aitchison geometry (Pawlowsky-Glahn and Egozcue 2001) that allows to analyze compositions in the usual Euclidean geometry.

The focus in this paper is on isometric log-ratio (ilr) coordinates (Egozcue et al. 2003), which allow to express a composition $\varvec{x}\in \mathcal {S}^D$ in the real space ${{\mathbb {R}}}^{D-1}$. A particular choice for ilr coordinates is

1 $$\begin{aligned} z_j=\mathrm{ilr}_j(\varvec{x})=\sqrt{{D-j} \over D-j+1} \ln \frac{x_j}{\root D-j \of {\prod _{k=j+1}^Dx_k}},\quad j=1, \ldots , D-1, \end{aligned}$$

and the coordinates $\varvec{z}=(z_1,\ldots ,z_{D-1})$, indeed, correspond to an orthonormal basis in ${{\mathbb {R}}}^{D-1}$ (Egozcue et al. 2003). The particular choice of the ilr coordinates in (1) allows for an interpretation of the first coordinate $z_1$, as that one expressing all relative information about part $x_1$, since $x_1$ is not included in any other ilr coordinate.

The definition of ilr coordinates (1) reveals that geometric means of (subsets of) the parts are involved. Note that the geometric mean of $\varvec{x}$ can also be expressed as

$$\begin{aligned} g_m(\varvec{x})={\left( \prod ^{D}_{j=1}x_j \right) }^{1 / D} = \text{ exp }\left( \frac{1}{D}\sum _{j=1}^D\ln x_j\right) \end{aligned}$$

involving the arithmetic mean of the log-transformed values. It is well known that the arithmetic mean is sensitive to data outliers (Maronna et al. 2006). Consequently, also data imprecision in one or some compositional parts (that are usually measured without respecting the relative nature of compositional data), or detection limit problems, may act like outliers and lead to a distortion of the geometric mean. The resulting ilr coordinates will suffer from data quality problems, and subsequent analyses based on these coordinates can be biased.

This unwanted effect is investigated here under the terminology of error propagation, where the effect of the errors on the output of a function is analyzed. Propagation of error can be performed by a calculus-based approach, or by simulation studies. A calculus-based approach makes use of the Taylor series expansion and calculates the first two statistical moments of the error of output, the mean and the variance, under the assumption that the errors are statistically independent (Ku 1966). With few exceptions, almost all analyses of error propagation with the calculus-based approach use the first-order Taylor approximation, and neglect the higher order terms (Birge 1939). This approach is briefly reviewed in Sect. 2. Section 3 starts with a motivating example about the effect of the errors on ilr coordinates and applies the concept of Taylor approximation to error propagation in the simplex. While this is done in a general form for any function (transformation), particular emphasis is given to error propagation for ilr coordinates that cause one source of distortion of outputs in practical geochemical problems (Filzmoser et al. 2009b).

Determining error propagation only for the first two moments is unsatisfactory, because it would also be interesting how the data structure is changed in the case of data problems like detection limits or imprecision of the measurements. Thus, simulation-based methods for error propagation are considered as well. The Monte Carlo method is adaptable and simple for the propagation of errors (Feller and Blaich 2001; Cox and Siebert 2006), and various applications of this method can be found (Liu 2008). The simulation-based approach in Sect. 4 makes use of a practical data set and shows the effect of imprecision and detection limit effects on the ilr coordinates. The interest lies particularly in error propagation on the first ilr coordinate, because this contains all relative information about the first compositional part, and on error propagation on all ilr coordinated jointly, because they contain the full multivariate information. The final Sect. 5 discusses the findings and concludes.

## Error Propagation in the Standard Euclidean Geometry

Consider a *p*-dimensional random variable $\varvec{x}=(x_1,\ldots ,x_p)$, and a function $f~:~{{\mathbb {R}}}^p \rightarrow {{\mathbb {R}}}$ that gives the output *y* as a result of $y=f(\varvec{x})$. The propagation of the errors of each variable through the function *f* on the output can be derived using Taylor approximation (Ku 1966). This yields a linear approximation of the function *f* by the tangent plane where the slopes in $x_1, \ldots , x_p$ are described by the partial derivatives $\frac{\partial y}{\partial x_1}, \ldots , \frac{\partial y}{\partial x_p}$ at a single point. One can express the random variables $\left( x_1, \ldots , x_p\right)$ as the sum of their expected values $\varvec{\mu }=\left( \mu _1, \ldots , \mu _p\right)$ and random deviations from the expected value $\varvec{\epsilon }=\left( \varepsilon _1, \ldots , \varepsilon _p\right)$, so that $\varvec{x}= \varvec{\mu }+ \varvec{\epsilon }$, assuming that the errors have mean zero. Taking the first-order Taylor approximation of $f(\varvec{x})$ results in

2 $$\begin{aligned} y= & {} f(x_1,\ldots ,x_p) =f(\mu _1+\epsilon _1, \ldots , \mu _p+\epsilon _p)\nonumber \\\approx & {} f(\mu _1,\ldots ,\mu _p)+\left[ \frac{\partial {f}}{\partial {x_1}}(\mu _1)\right] \epsilon _1+ \cdots +\left[ \frac{\partial {f}}{\partial {x_p}}(\mu _p)\right] \epsilon _p. \end{aligned}$$

In the framework of error propagation, it is common to assume that $(x_1, \ldots , x_p)$ follow a known distribution, in most cases, a multivariate normal distribution (Ku 1966). If the distribution is known, the partial derivatives are evaluated at the true means, if not, the sample averages are used for the estimation. The approximation in Eq. (2) can now be used to calculate mean and variance of *y*, which both depend on the function *f*. The second central moment, the variance Var(*y*), describes the uncertainty, which is mainly used to investigate the effect of error propagation and is given as

3 $$\begin{aligned} \text{ Var }(y)\approx \sum ^{p}_{j=1}\left( \frac{\partial {f}}{\partial {x_j}}(\mu _j)\right) ^{2}E({\epsilon _j}^{2})+ \mathop {\sum \sum }_{j\ne k}\left( \frac{\partial {f}}{\partial {x_j}}(\mu _j)\right) \left( \frac{\partial {f}}{\partial {x_k}}(\mu _k)\right) E({\epsilon _j \epsilon _k}).\nonumber \\ \end{aligned}$$

Equation (3) reveals how the variability of the output *y* depends on the errors and on the function *f*.

## Error Propagation on the Simplex

**Fig. 1:**
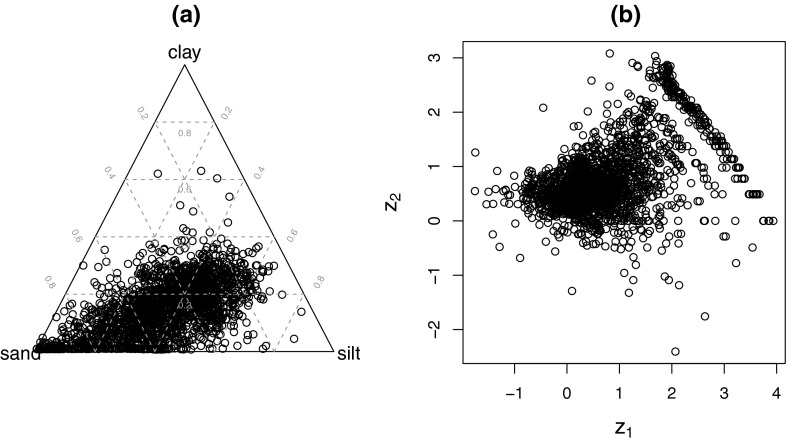
Composition of sand, silt, and clay in agricultural soils of Europe. Ternary diagram **a**, representation in ilr coordinates **b**

As a motivating example, the composition of sand, silt, and clay in agricultural soils in Europe is considered. The data are reported in Reimann et al. (2014). From the ternary diagram (Fig. 1a), it can be seen that the clay concentrations can be very small, but data artifacts are not immediately visible. The resulting ilr coordinates $z_1$ and $z_2$ are shown in Fig. 1b. Here, the small clay values are visible in form of a band that deviates clearly from the joint data structure. In fact, small values of clay have been rounded in the laboratory, which causes already a distortion of the multivariate data structure. Thus, the imprecision here is visible as a rounding effect in the part clay. Variables with values below a detection limit can result in similar artifacts, since usually the values below detection are set to some constant, like 2/3 times the values of the detection limit (Martín-Fernández et al. 2003). This is still the usual practice in geosciences rather than employing more sophisticated algorithms for their imputation (Martín-Fernández et al. 2012).

Similar as in Sect. 2, error propagation is derived for a general function using first-order Taylor approximation. However, since this is directly done on the simplex, also the Taylor approximation needs to be done on the simplex. The theoretical background for the differential calculus on the simplex can be found in Barceló-Vidal and Martín-Fernández (2002) and Barceló-Vidal et al. (2011). Here, the tools necessary to carry out the Taylor approximation are recalled.

Let $f:U\rightarrow {{\mathbb {R}}}^m$ be a vector-valued function defined on a subset $U\subset {{\mathbb {R}}}^D_+$. Let $\underline{U}=\{\mathcal {C}(\varvec{w}), \varvec{w}\in U \}$, the compositional closure of *U*, be a subset of $\mathcal {S}^D$. If *f* is scale invariant, that is $f(\varvec{w})=f(k\varvec{w})$ for any $k>0$, it induces a vector-valued function $\underline{f}:\underline{U}\rightarrow {{\mathbb {R}}}^m$. It suffices to define

$$\begin{aligned} \underline{f}(\varvec{x})=f(\varvec{w}),\quad \forall \varvec{w}\in U \text{, } \end{aligned}$$

where $\mathcal {C}(\varvec{w})=\varvec{x}$ (Barceló-Vidal et al. 2011). The function $\underline{f}$ is $\mathcal {C}$-differentiable at $\varvec{x}\in \underline{U}$, if there exists an $m \times D$ matrix $\varvec{A}=( a _{ij})$, satisfying $\varvec{A}\varvec{1}_D=\varvec{0}_m$ (defining a linear transformation from ${{\mathbb {R}}}^D$ to ${{\mathbb {R}}}^m$), such that

$$\begin{aligned} \lim _{\varvec{u}\xrightarrow {\mathcal {C}} \varvec{n}}\ \frac{\Vert \underline{f}(\varvec{x}\oplus \varvec{u}) - \underline{f}(\varvec{x}) - \varvec{A}~ \text{ ln } \varvec{u}\Vert }{\Vert \varvec{u}\Vert _A}=0 \end{aligned}$$

for $\varvec{u}\in \underline{U}$, where $\varvec{1}_D=(1, \ldots ,1)$ with length *D*, and $\varvec{0}_m=(0,\ldots ,0)$ with length *m*. Note that $\varvec{n}=\mathcal {C}(1, \ldots , 1)$ is the neutral element of $(\mathcal {S}^D, \oplus )$ and $\varvec{u}\xrightarrow {\mathcal {C}} \varvec{n}$ denotes that $\varvec{u}$ converges to $\varvec{n}$ on the simplex. From the definitions above, the first-order Taylor approximation of a real-valued function $\underline{f}$ can be written as

4 $$\begin{aligned} \underline{f}(\varvec{x}\oplus {\varvec{u}}) \approx \underline{f}(\varvec{x})+\sum ^{D}_{j=1} \text{ ln } (u_j) \left[ \frac{\partial _\mathcal {C} \underline{f}}{\partial {x_j}}(\varvec{x}) \right] , \end{aligned}$$

where the $\mathcal {C}$-derivative of $\underline{f}$ exists and is equal to

5 $$\begin{aligned} \frac{{\partial }_\mathcal {C} \underline{f}}{\partial x_j}(\varvec{x})=x_j\left( \frac{\partial \underline{f}}{\partial x_j}(\varvec{x}) -\sum ^{D}_{i=1}x_i\frac{\partial \underline{f}}{\partial x_i}(\varvec{x})\right) \quad \text{ for } j=1,\ldots , D. \end{aligned}$$

Given a *D*-part composition $\varvec{x}=(x_1, \ldots , x_D) \in S^D$, which can be expressed as a perturbation of its center $\varvec{\mu }=\left( \mu _1,\ldots ,\mu _D\right)$ (Pawlowsky-Glahn and Egozcue 2002) and random deviations $\varvec{\epsilon }=(\epsilon _1, \ldots , \epsilon _D)$ from the center, so that $\varvec{x}=\varvec{\mu }\oplus \varvec{\epsilon }$, then (4) can be rewritten as

6 $$\begin{aligned} \underline{f}(\varvec{\mu }\oplus {\varvec{\epsilon }})\approx \underline{f}(\varvec{\mu })+\sum ^{D}_{j=1} \text{ ln } (\epsilon _j) \left[ \frac{\partial _\mathcal {C} \underline{f}}{\partial {\mu _j}}(\varvec{\mu }) \right] . \end{aligned}$$

One can proceed as in Sect. 2 to derive the variance of the components of $\underline{f}(\varvec{\mu }\oplus {\varvec{\epsilon }})$. Similar as for the Taylor expansion (2) from Sect. 2, also here, the approximation is valid just for small perturbations. Moreover, in contrast to the previous case, the error is now multiplicative. Although this fits well with the nature of compositional data, particularly with their scale invariance, in practice, error terms are often additive (van den Boogaart et al. 2015). This fact should be considered for an error propagation analysis of compositional data.

In the case of ilr coordinates, however, the investigation of the error propagation simplifies. By considering (6) with ilr coordinate $ilr_i(\varvec{x})$ as *i* th component of $\underline{f}$

7 $$\begin{aligned} \frac{\partial _c \mathrm{ilr}_i}{\partial {\mu _j}} = \left\{ \begin{array}{ll} 0 &{}\quad \text{ if } j < i, \\ \sqrt{{D-i} \over {D-i+1}} &{} \quad \text{ if } j=i, \\ -\sqrt{{D-i} \over {D-i+1}}\frac{1}{D-i} &{}\quad \text{ if } j > i,\\ \end{array} \right. \end{aligned}$$

where $i=1, \ldots , D-1$. This corresponds exactly to a logcontrast (Aitchison 1986) of the *i* th ilr coordinate of the compositional error $\varvec{\epsilon }$, and consequently

$$\begin{aligned} \mathrm{ilr}_i(\varvec{x})=\mathrm{ilr}_i(\varvec{\mu }\oplus \varvec{\epsilon })=\mathrm{ilr}_i(\varvec{\mu })+\mathrm{ilr}_i(\varvec{\epsilon }),\ i=1,\ldots ,D-1. \end{aligned}$$

In the context of error propagation this shows that the ilr coordinates are additive with respect to multiplicative errors. On the other hand, for other forms of errors, a non-linear behavior can be expected. This issue is further investigated within the simulation study in Sect. 4.

In addition, this leads to an alternative verification of the linearity of ilr coordinates

$$\begin{aligned} \varvec{z}=\mathrm{ilr}(\varvec{x})=\mathrm{ilr}(\varvec{\mu }\oplus \varvec{\epsilon })=\mathrm{ilr}(\varvec{\mu })+\mathrm{ilr}(\varvec{\epsilon }), \end{aligned}$$

that is commonly shown directly with the definitions from Sect. 1. Even more, ilr coordinates represent an isometry, which means that all metric concepts in the simplex are maintained after taking the ilr coordinates (Pawlowsky-Glahn et al. 2015). The variance can now be considered component-wise, for example for the *j* th component $z_j$ of $\varvec{z}$ one obtains

$$\begin{aligned} \text{ Var }(z_j)=\text{ Var }(\mathrm{ilr}_j(\varvec{x}))=\text{ Var }(\mathrm{ilr}_j(\varvec{\epsilon })). \end{aligned}$$

This variance can be expressed by log-ratios of the compositional parts, as shown in Fišerová and Hron (2011) as

8 $$\begin{aligned} \text{ Var }(z_j)= & {} A - B \quad \text{ with } \nonumber \\ A= & {} \frac{1}{D - j +1 } \sum \limits _{k=j+1}^{D} \text{ Var }\left( \ln \frac{\epsilon _j}{\epsilon _k}\right) , \nonumber \\ B= & {} \frac{1}{2(D-j)(D -j+1)} \sum \limits _{k=j+1}^{D}\sum \limits _{l=j+1}^{D}\text{ Var }\left( \ln \frac{\epsilon _k}{\epsilon _l}\right) . \end{aligned}$$

The contributions of log-ratio variances in this linear combination are clearly higher for terms in *A* that include $\epsilon _j$, and lower for terms in *B* where $\epsilon _j$ is not involved, and their magnitude depends on the number of parts *D*. In particular, if *D* is large and contamination (imprecision, detection limit problem) is expected only in one compositional part, the effect on the variance of $z_j$ will be small. Note, however, that for a multivariate analysis, the focus is in all coordinates $z_1,\ldots ,z_{D-1}$ simultaneously, and thus, it is not so straightforward to investigate the effect, since there may also be dependencies among the error terms. There is a simple exception: suppose that an error is to be expected only in log-ratios with one compositional part. From a practical perspective, it would then appear that only one compositional part is erroneous. If this part is taken as the first one, the ilr coordinates from Eq. (1) will allow to assign this error exclusively to $z_1$, but not to the other coordinates.

Besides investigating the variance of the coordinates, it is also important to know how the errors affect distances between different compositions, that is between observations of a compositional data set, and how the multivariate data structure is affected. All these aspects will be investigated in more detail by simulations in the next section.

## Simulation-Based Investigations of Error Propagation

For a simulation-based analysis of error propagation, a real data set is used, namely the GEMAS data mentioned in Sect. 1, described in Reimann et al. (2014). More than 2000 samples of agricultural soils have been analyzed in an area covering 5.6 million km$^2$ of Europe across 33 countries, and for the simulations, the concentrations of the elements Al, Ba, Ca, Cr, Fe, K, Mg, Mn, Na, Nb, P, Pb, Rb, Si, Sr, Ti, V, Y, Zn, and Zr are considered. Precision or detection limit problems of these elements are rather small or even not existing (Reimann et al. 2014), and thus, these elements form a good base for carrying out simulations where contamination is artificially introduced in the form of imprecision and detection limit problems.

Denote the resulting compositional data matrix by $\varvec{X}$, where the observations are forming the rows and the above-mentioned compositional parts the columns. The number of observations is $n=2107$, and the number of parts is $D=20$. The cells of the matrix $\varvec{X}$ are denoted as $x_{ij}$, for $i=1,\ldots ,n$ and $j=1,\ldots ,D$.

In the simulations, problems with detection limit and imprecision are reproduced as follows:

- *Detection limit* (*DL*) Set all observations $x_{ij}$ of the *j* th part to the value
  9 $$\begin{aligned} x_{ij}^* = \left\{ \begin{array}{ll} \frac{2}{3}\text{ DL }_j &{} \quad \text{ if } x_{ij}\le \text{ DL }_j \\ x_{ij} &{} \quad \text{ otherwise },\\ \end{array} \right. \end{aligned}$$
  where $i=1,\ldots , n$, and $\text{ DL }_j$ is taken as some quantile of that part.
- *Imprecision rate* (*IR*) A noise term $\epsilon _{ij}$ is added to each observation $x_{ij}$, where the noise depends on the magnitude of the observation and follows a uniform distribution. Thus, the values $x_{ij}$, $i=1,\ldots ,n$, are set to
  10 $$\begin{aligned} x_{ij}^*=x_{ij} + \epsilon _{ij}, \;\; \epsilon _{ij}\sim \mathcal {U}(-\alpha _j x_{ij}, \alpha _j x_{ij}), \end{aligned}$$
  where $\alpha _j>0$ defines the imprecision rate of the *j* th part, and the resulting simulated value $x_{ij}^*$ must be positive. Note that this contamination is not additive but multiplicative, since
  $$\begin{aligned} x_{ij}^*=x_{ij}(1 + \gamma _{j}), \;\; \gamma _{j}\sim \mathcal {U}(-\alpha _j, \alpha _j). \end{aligned}$$
  Thus, this contamination scheme corresponds to the error model of the previous section, while contamination by a detection limit introduces a non-linear effect.

As mentioned previously, the main interest is the investigation of error propagation for ilr coordinates. If the *i* th row of $\varvec{X}$ is denoted by $\varvec{x}_i$, then the ilr coordinates are obtained by Eq. (1), leading to the values $\varvec{z}_i=(z_{i1},\ldots ,z_{i,D-1})$. The complete $n\times (D-1)$ matrix of coordinates is denoted by $\varvec{Z}$, with cells $z_{ij}$.

**Fig. 2:**
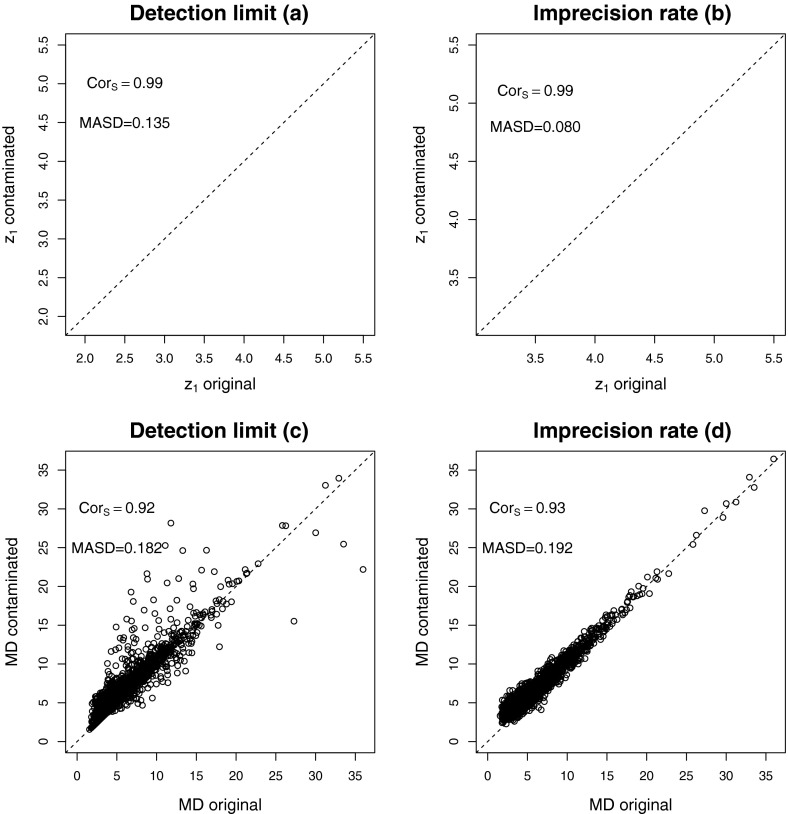
Effect of the DL and IR contamination on the first ilr coordinate (**a** and **b**), and on all ilr coordinates jointly (**c** and **d**)

As an illustrative example the last ten parts of the composition are picked, and contaminated with errors. A detection limit problem is imitated, by choosing $\text{ DL }_j$ as the 0.25-quantile in each of these components, and setting the values in these parts according to Eq. (9). The results are shown in the left panels of Fig. 2: the upper panel shows the first ilr coordinate of the original versus the contaminated data. One can see clear distortions in form of deviations from the main structure, but also in the form of nonlinearities. For a clearer picture of the multivariate data structure, the Mahalanobis distances of all ilr coordinates for the original and contaminated data are presented in the lower panel of Fig. 2. The Mahalanobis distance (MD) for the *i* th composition expressed in coordinates is

11 $$\begin{aligned} \text{ MD }(\varvec{z}_i) =\sqrt{(\varvec{z}_i - \varvec{t}_{\varvec{z}})'\varvec{C}_{\varvec{z}}^{-1}(\varvec{z}_i - \varvec{t}_{\varvec{z}})}, \quad \text{ for } i=1, \ldots , n, \end{aligned}$$

where $\varvec{t}_{\varvec{z}}$ and $\varvec{C}_{\varvec{z}}$ are robust estimators of location and covariance of the ilr coordinates $\varvec{Z}$, respectively. For reasons of comparability, the Mahalanobis distances for the contaminated data are computed with the estimators $\varvec{t}_{\varvec{z}}$ and $\varvec{C}_{\varvec{z}}$ based on the uncontaminated data. Plugging in robust estimators is essential, since they guarantee that the Mahalanobis distance estimation is not spoiled by single outliers, but based on the data majority. For this purpose, the minimum covariance determinant (MCD) estimator is used (Rousseeuw and Van Driessen 1999).

The right panel of Fig. 2 shows the results of a simulated precision problem. Again, the last ten parts are contaminated, $\alpha _j$ is set to 0.25 for these parts, and Eq. (10) is applied. The upper panel compares the first ilr coordinates for the original and distorted data. Since the contamination is symmetric in each part, the outcome is also relatively symmetric around the line of 45 degrees. The comparison of the Mahalanobis distance shows that those distances for the contaminated data increase, in general.

The above example already provides an idea about possible choices of measures for quantifying the resulting error. The focus is on the first ilr coordinate as well as on all coordinates jointly in terms of Mahalanobis distances, and the original data will be compared with the contaminated data.

Denote the values of the first ilr coordinate by $\varvec{z}_{0.1}=(z_{11},\ldots ,z_{n1})$, and the corresponding contaminated version by $\varvec{z}_{0.1}^*=(z_{11}^*,\ldots ,z_{n1}^*)$. The two vectors are compared by:

- Spearman rank correlation, expressed as
  12 $$\begin{aligned} \text{ Cor }_S(\varvec{z}_{0.1},\varvec{z}_{0.1}^*)= \frac{\text{ Cov }(R(\varvec{z}_{0.1}), R(\varvec{z}_{0.1}^*))}{\sqrt{\text{ Var }(R(\varvec{z}_{0.1}))}\sqrt{\text{ Var }(R(\varvec{z}_{0.1}^*))}}, \end{aligned}$$
  where $R(\cdot )$ gives the ranks of its argument vector.
- Mean absolute scaled deviation (MASD), defined as
  13 $$\begin{aligned} \text{ MASD }(\varvec{z}_{0.1}, \varvec{z}_{0.1}^*)=\frac{1}{n}\sum \limits _{i=1}^{n} \frac{\left| z_{i1} - z_{i1}^*\right| }{\sqrt{\text{ Var }(\varvec{z}_{0.1})}}. \end{aligned}$$

The Spearman rank correlation coefficient measures the monotone relation between the uncontaminated and contaminated coordinates; a value of one would refer to the same ordering of the values of the coordinates. On the other hand, MASD is more strict and evaluates the error in reproducing the values of the coordinate. Note that the scaling in MASD by the variance is used to allow for a comparison of the corresponding first ilr coordinates if the parts in the data matrix are permuted.

Similar measures for comparison are proposed in the multivariate case. Denote by $\text{ MD }(\varvec{Z})$, the vector of the Mahalanobis distances $\text{ MD }(\varvec{z}_i)$, for $i=1,\ldots ,n$, see Eq. (11), and by $\text{ MD }(\varvec{Z^*})$ the corresponding contaminated version, with entries $\text{ MD }(\varvec{z}_i^*)$. Then, the Spearman rank correlation coefficient $\text{ Cor }_S(\text{ MD }(\varvec{Z}),\text{ MD }(\varvec{Z^*}))$ investigates if the overall ordering in the multivariate data structure, represented in coordinates, is maintained. A mean absolute scaled deviation (MASD) measure relates to the Mahalanobis distances

14 $$\begin{aligned} \text{ MASD }(\text{ MD }(\varvec{Z}),\text{ MD }(\varvec{Z^*}))=\frac{1}{n}\sum \limits _{i=1}^{n} \frac{\left| \text{ MD }(\varvec{z}_{i}) - \text{ MD }(\varvec{z}_{i}^*)\right| }{Q_{0.5}(\text{ MD }(\varvec{Z}))}. \end{aligned}$$

The scaling is done by the 0.5 quantile (median) of the Mahalanobis distances of $\varvec{Z}$ to allow for comparability of subcompositions with different numbers of parts. This measure, thus, indicates the error in reproducing the multivariate data structure. As mentioned previously, the Mahalanobis distances $\text{ MD }(\varvec{Z^*})$ are based on the estimates of location $\varvec{t}_{\varvec{z}}$ and covariance $\varvec{C}_{\varvec{z}}$ of the matrix $\varvec{Z}$, see Eq. (11), leading to a MASD value of zero for observations which have not been changed.

These measures have been computed for the example shown in Fig. 2 to get an idea about the meaning of the magnitude of these values. The Spearman rank correlation is in all cases clearly above 0.9, in spite of the deviations of some points. The scaled distances MASD for the first ilr coordinates are lower that those for all coordinates jointly (Mahalanobis distances).

### Simulation 1: One Uncontaminated, 1 to 19 Contaminated Parts

Start with the first column $\varvec{x}_{0.1}$ of the composition $\varvec{X}$, and add step-by-step another column. After the $(k-1)$-st step, one ends up with the subcomposition $\varvec{X}_k=(\varvec{x}_{0.1},\varvec{x}_{0.2},\ldots ,\varvec{x}_{0.k})$, where $k=2,\ldots ,20$. A contaminated version is generated by contaminating all parts except the first one; this yields $\varvec{X}_k^*=(\varvec{x}_{0.1},\varvec{x}_{0.2}^*,\ldots ,\varvec{x}_{0.k}^*)$. Then, the ilr coordinates are computed from $\varvec{X}_k$ and $\varvec{X}_k^*$, and the measures $\text{ Cor }_S$ and MASD are calculated for the first coordinates and for all coordinates jointly in terms of Mahalanobis distances.

The number of simulation replications is 100. In each replication, the parts of the original composition are permuted. In that way, the first (uncontaminated) part changes, but also the sequence of the parts that are added changes. All simulations are done for the contamination in the form of detection limit (DL) and for imprecision (IR). In the first case, the value DL$_j$ of the detection limit is taken as the 0.25 quantile, see Eq. (9), while in the latter case, the imprecision rate is taken as $\alpha _j=0.25$, see Eq. (10).

**Fig. 3:**
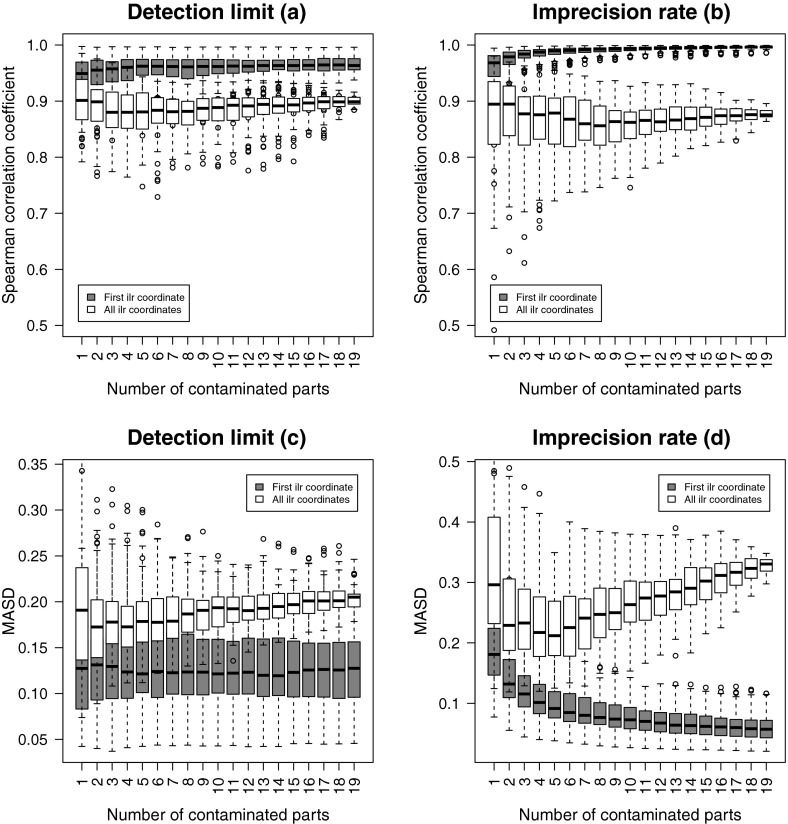
One uncontaminated part, and 1–19 contaminated parts added. Univariate and multivariate structural changes between the original and contaminated ilr coordinates with increasing number of contaminated parts in case of DL (**a** and **c**) and IR (**b** and **d**)

The results are presented by boxplots in Fig. 3. The left panels show the outcome for the detection limit simulations, and the right panels show the results of the imprecision simulations. The upper figures show the comparison of original versus contaminated versions in terms of Spearman correlations, while the lower figures compare in terms of MASD. The grey boxplots compare the first ilr coordinates, while the white boxplots summarize the Mahalanobis distances of all joint coordinates. The plots allow to compare the impact of an increasing number of contaminated parts (horizontal axis). Although the amount of contamination is quite high, the correlations reveal that the covariance structure of the multivariate data is basically preserved. In particular, the comparison of the first ilr coordinates leads to a remarkably high correlation, which is quite stable with an increasing number of parts (for DL), and even improving in the case of IR. This means that additional parts coupled with a symmetric contamination scheme, as in the case of IR, still provide important and useful information that stabilizes the first ilr coordinate. The MASD results for the first ilr coordinate are quite stable in the case of DL, while in the IR case with increasing number of parts an improvement is observed.

The picture is somewhat different when comparing all ilr coordinates jointly. The Spearman correlation is clearly lower, and it gets more stable with an increasing number of parts. In the case of DL, the MASD measure is nearly constant with an increasing number of parts, while for IR first, a decline is observed, but then a clear increase. It is, however, surprising that the Mahalanobis distances do not change more drastically, given that the amount of contamination is relatively high.

### Simulation 2: 10 Uncontaminated, 1 to 10 Contaminated Parts

**Fig. 4:**
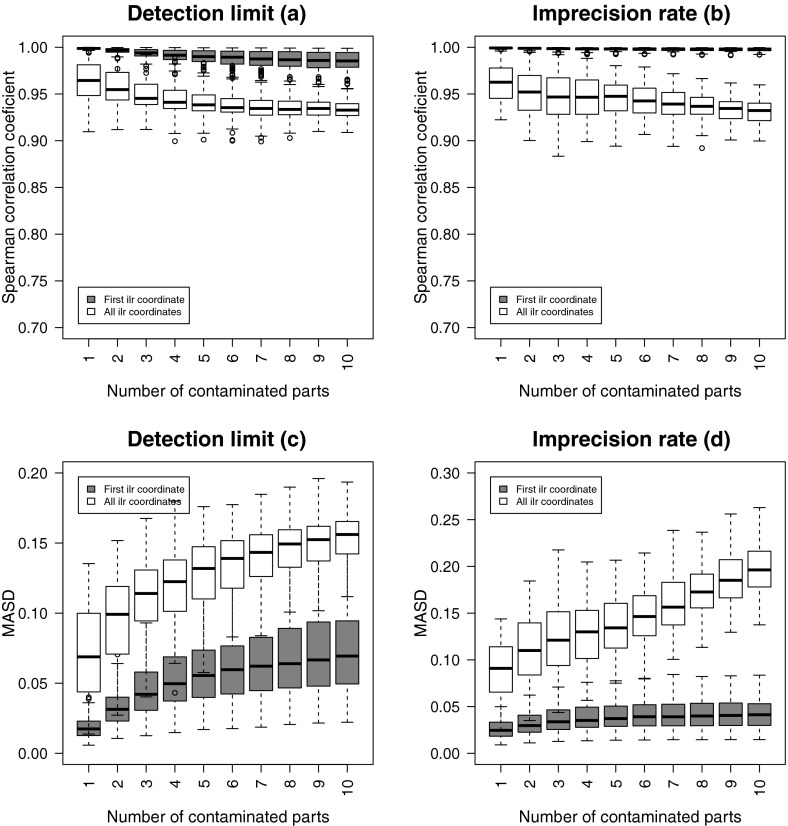
Ten uncontaminated parts, and 1 to 10 contaminated parts added. Univariate and multivariate structural changes between the original and contaminated ilr coordinates with increasing number of contaminated parts in the case of DL (**a** and **c**) and IR (**b** and **d**)

In a further simulation experiment, a block of ten compositional parts is fixed and left uncontaminated. Step-by-step, a contaminated part is added, until all ten remaining (contaminated) parts have been included. The comparison is done in the same way as before. The simulation is repeated 100 times, and the parts are randomly permuted for each replication. Thus, the uncontaminated block changes, but also the contaminated parts differ from simulation to simulation. The results are shown in Fig. 4.

Basically, a similar impression can be observed as in Fig. 3. For the first ilr coordinates, the correlations are now very close to one, and the values of MASD, although increasing slightly with increasing number of contaminated parts, are close to zero. Therefore, having good data quality for a major part of the data set is a good protection against poor data quality in additional parts—at least for the first ilr coordinate. The multivariate data structure is well maintained in terms of ordering, expressed by the Spearman rank correlations, which are still clearly above 0.9. The MASD values for the Mahalanobis distances now increase for DL as well as for IR, with an increasing number of parts, but they are lower than in the previous simulation.

### Simulation 3: Changing the Amount of Contamination

**Fig. 5:**
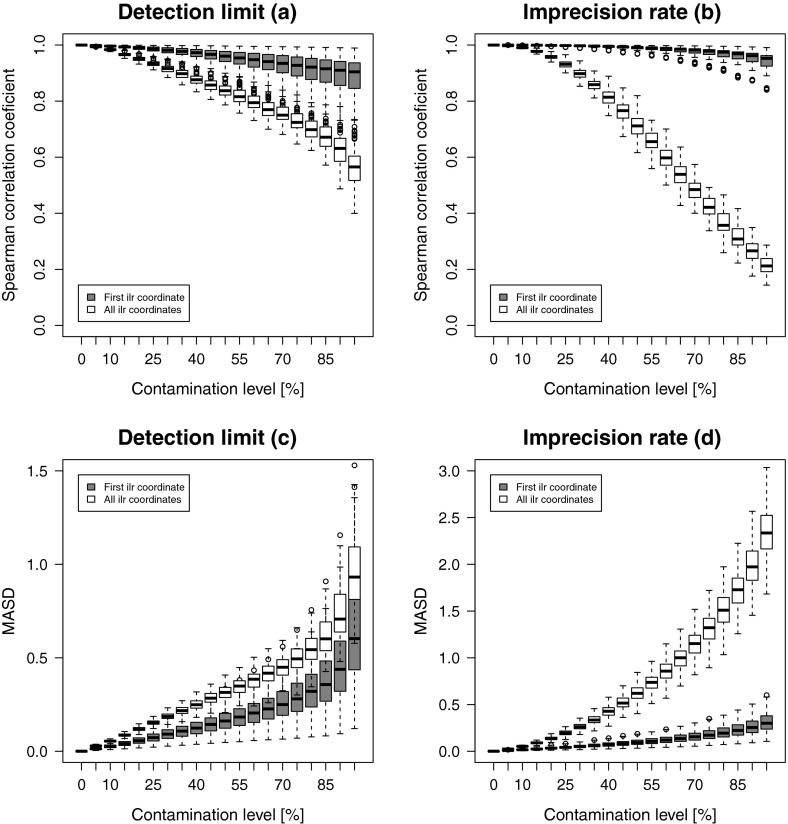
Ten uncontaminated parts, and 10 contaminated parts added. Univariate and multivariate structural changes between the original and contaminated ilr coordinates with increasing amount of contamination in case of DL (**a** and **c**) and IR (**b** and **d**)

In the previous simulations, the amount of contamination is fixed. Here, the effect of changing the amount of contamination is investigated. For that purpose, ten parts are selected randomly to leave them uncontaminated, while the remaining ten parts are contaminated by the same amount: in the case of DL contamination, the value DL$_j$ is varied from the 0.05-quantile to the 0.95-quantile; for IR contamination, the imprecision rate $\alpha _j$ is varied from 0.05 to 0.95. Note that the imprecision in real studies can be much higher, in particular for small concentrations (Reimann et al. 2014). Fig. 5 summarizes the outcome of the simulations, where again 100 replications were performed.

The resistance against contamination of the first ilr coordinate is remarkable. Both the correlation and the MASD report relatively small deviations, even for very high amounts of contamination. Contamination according to DL has more effect than that based on imprecision. This is different when looking at the multivariate data structure, expressed by the joint coordinates. The correlations get severely low, and also the MASD increases rapidly. The effect for IR contamination is more severe than that for DL. A MASD value of one means that the average change of the Mahalanobis distances before and after contamination is as large as the median Mahalanobis distance, and thus, this would correspond to a substantial change in the multivariate data structure.

### Simulation 4: Changing the Number of Observations

**Fig. 6:**
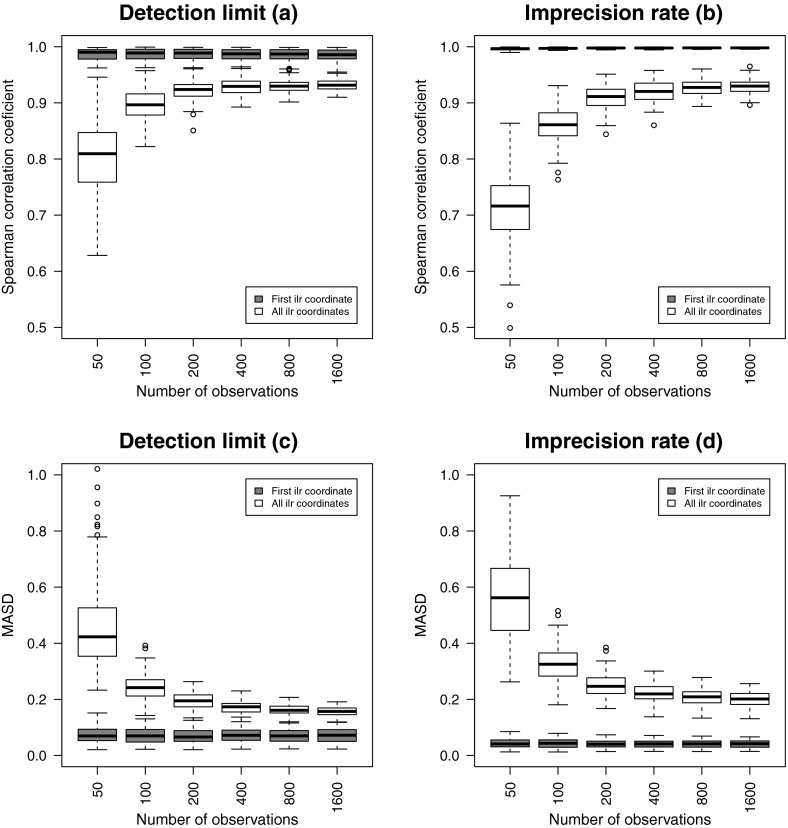
Ten uncontaminated parts, and ten contaminated parts added. Univariate and multivariate structural changes between the original and contaminated ilr coordinates with varying number of observations in the data set; the contamination level is fixed with 25 %; DL (**a** and **c**) and IR (**b** and **d**)

In a final simulation, the effect of the number of observations in the data set, which has been fixed before with all available observations (i.e., more than 2000), is analyzed. As before, ten parts are randomly selected and not modified, and the remaining ten parts are contaminated at a level of 25 %, that is for DL contamination 25 % of values below detection limit in each of these parts, and for IR contamination $\alpha _j=0.25$ for these parts. The results in Fig. 6 for the 100 simulations show that there is no visible effect for the first ilr coordinate. However, the multivariate structure suffers severely if the number of observations is smaller than 100.

## Discussion and Conclusions

To many practitioners, it looks almost obvious that geometric means, as they are used in log-ratio approaches, may cause instabilities due to the involved products of the data values. Even worse, measurement errors could be propagated by the use of geometric means. This problem is investigated in more detail, by focusing on the most important log-ratio approach based on ilr coordinates (Pawlowsky-Glahn and Buccianti 2011; Pawlowsky-Glahn et al. 2015).

**Fig. 7:**
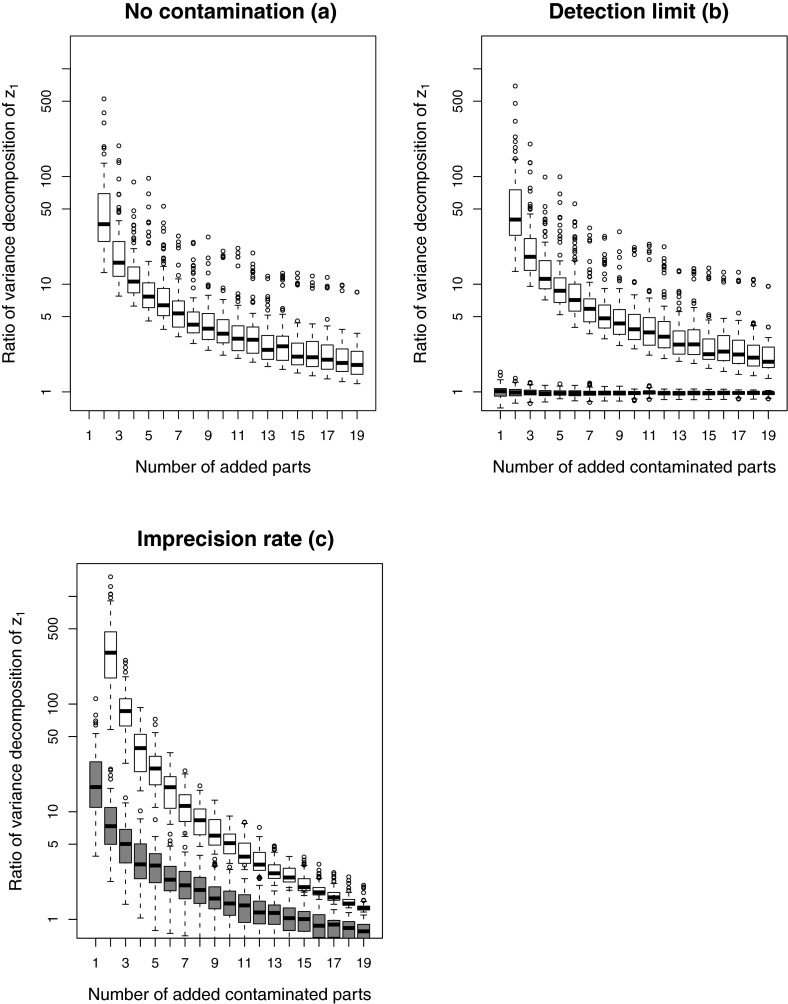
Variance decomposition results: The *light* boxplots show the ratios *A* / *B* according to Eq. (8) for the simulation scheme in Sect. 4.1. The *dark* boxplots compare the ratio of *A*-contaminated (either DL or IR) to *A*-uncontaminated. No contamination (**a**), detection limit (**b**), and imprecision rate (**c**)

In a first attempt, the classical theory of error propagation has been formulated on the simplex, the sample space of compositional data. While this gets complex if any transformation function would be considered, the results are straightforward when using ilr coordinates because of their linearity. It has been shown that the variance of an ilr coordinate is just the variance of the same ilr coordinate of the random deviations from the center. Using Eq. (8), it can be seen which terms contribute by which magnitude to this variance. For non-linear contamination schemes, these variance contributions cannot be computed from the random errors, but they have to be computed directly from the ilr coordinate. This has been done for the simulation scheme outlined in Sect. 4.1 for the first ilr coordinates $z_1$ of the uncontaminated data, the data contaminated by a detection limit, and contaminated by the imprecision rate. The resulting variance contributions are shown in Fig. 7 in the form of ratios *A* / *B* according to Eq. (8) as non-colored boxplots. With increasing number of parts, the term *B* (which does not involve variance contributions with log-ratios to $x_1$) gets more dominant. This can be seen in the uncontaminated case, as well as in the contaminated cases due to the inherent variability contained within the log-ratios of the remaining parts. Interestingly, detection limit contamination has almost no effect on the variance contributions *A* and *B* when compared to the uncontaminated case. This is also shown by the dark boxplots which represent the ratios of *A*-contaminated to *A*-uncontaminated. Only for contamination by the imprecision rate, the variance contributions are clearly higher compared to the uncontaminated case if the number of contaminated parts is low. For higher numbers of contaminated parts, the variance contributions are about the same.

Further investigations have been carried out through simulation experiments. The contamination is studied in terms of mimicking a detection limit problem, and in terms of imprecision in form of a multiplicative factor. In all experiments it turned out that the structure of the first ilr coordinate can almost not be destroyed with poor data quality, except in the case of extremely high amounts of contamination. This is an interesting outcome, since due to the proposed formula (1) to derive the ilr coordinates, the first coordinates describes all relative information about the first compositional part (Fišerová and Hron 2011). Clearly, if the main interest is not in the first, but in another part, then this part is simply put to the first position. Note that the first coordinate is proportional to the corresponding centered log-ratio (clr) coefficient (Aitchison 1986) for this part (Fišerová and Hron 2011). Practitioners often explore just the structure of the resulting clr coefficients. For example, one can study the clr coefficients for the different chemical elements in maps, which is the compositional alternative to the traditional maps based on the absolute concentrations. Examples are shown in Reimann et al. (2014).

**Fig. 8:**
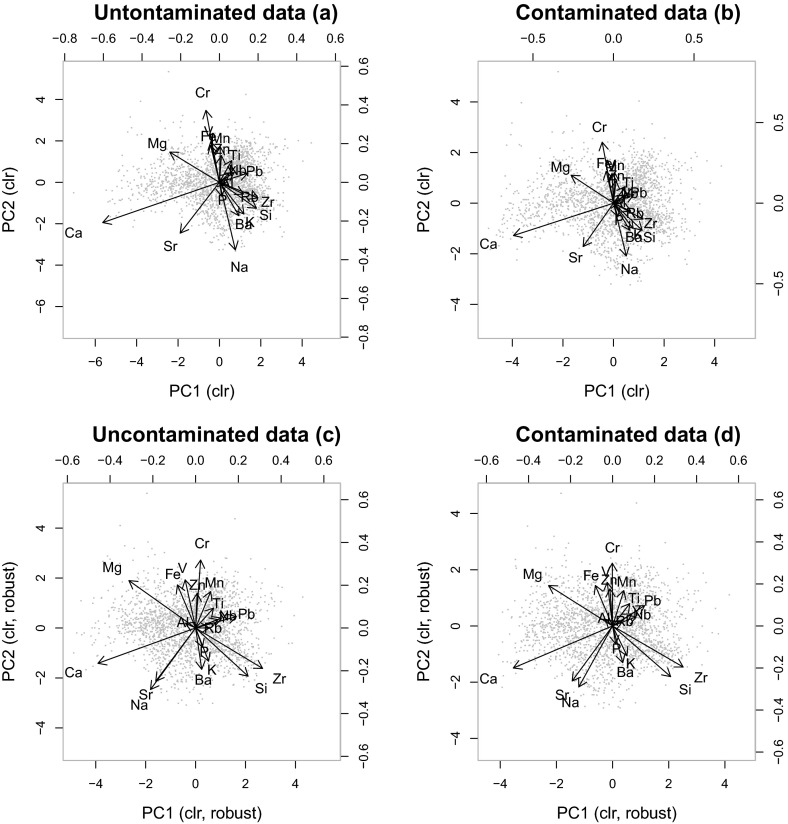
Biplots of the first two PCs based on the data shown in Fig. 2. Classical PCs of uncontaminated data (**a**), classical PCs of contaminated data (**b**), robust PCs of uncontaminated data (**c**), and robust PCs of contaminated data (**d**)

It is not studied, how the contamination of the first part effects the first ilr coordinate ($z_1$), because it is clear that the contamination would be immediately reflected in the first ilr coordinate, and any additional contamination in other parts would make things worse. Hence, variations of $z_1$ are only due to variations of $(x_2,\ldots ,x_D)$. It is, therefore, quite logical that the impact of DL or IR on $z_1$ remains limited, and that its growth decreases as *D* increases, due to compensation effects when computing $g_m(x_2,\ldots x_D)$.

Especially when applying multivariate statistical methods, such as principal component analysis or discriminant analysis, all ilr coordinates have to be analyzed jointly. Therefore, the effect of errors on the multivariate data structure is also investigated in the simulations. It depends very much on the setting if the multivariate data structure is destroyed by the contamination or not. If dimension increases, the effects of the contamination generally increase. It depends a lot on the contamination level if the multivariate data structure after contamination is still closely related to that before, but this also depends on the sample size of the data: higher numbers of observation (e.g., at least 100 in the data set used here) stabilize the results.

Consider again the example shown in Fig. 2, where 10 parts out of 20 have been contaminated at a level of 25 %. Here, the DL contamination scheme is considered. Figure 8 shows the biplots for the first two principal components (PCs): left panels for the uncontaminated data, right panels for the contaminated data. A comparison is also done with robust PCs (Filzmoser et al. 2009a), which are shown at the lower panels. While there is almost no difference visible between the uncontaminated and contaminated versions, there is a clear difference in the outcome of classical and robust principal component analysis. This shows that, although the MASD is around 0.18 (Fig. 2c), the outliers that are present in the data have a much stronger effect than the artificial contamination used here.

The overall conclusion of this paper is not that one does not have to care anymore about data quality issues. In contrary, good data quality is the basis of any sound statistical analysis. Rather, it should provide an answer to researchers who have a data set available, and who carefully think about which compositional parts to include in the analysis. Often, it is known which parts have precision problems, and sometimes even the level of imprecision is known. In addition, the amount of values below detection is known. Including such parts with moderate quality in the analysis will in general not have a major effect on a single (the first) ilr variable, and the effects will also be limited, in general, for the multivariate data structure.

The point why one should consider including as much information as possible in the analysis is because the reliable values of such parts with moderate data quality also contribute to the log-ratio analysis, and they might contain important and relevant information.
